# Translating Observations From Leishmanization Into Non-Living Vaccines: The Potential of Dendritic Cell-Based Vaccination Strategies Against *Leishmania*

**DOI:** 10.3389/fimmu.2018.01227

**Published:** 2018-06-04

**Authors:** Negar Seyed, Nathan C. Peters, Sima Rafati

**Affiliations:** ^1^Department of Immunotherapy and Leishmania Vaccine Research, Pasteur Institute of Iran, Tehran, Iran; ^2^Cumming School of Medicine, Snyder Institute for Chronic Diseases of Canada, University of Calgary, Calgary, Canada

**Keywords:** *Leishmania*, vaccine design, antigen persistence, effector T cells, long-term protection

## Abstract

Leishmaniasis is a health-threatening vector-borne disease in almost 90 different countries. While a prophylactic human vaccine is not yet available, the fact that recovery from leishmaniasis establishes lifelong immunity against secondary infection suggests that a vaccine is attainable. In the past, deliberate infection with virulent parasites, termed Leishmanization, was used as a live-vaccine against cutaneous leishmaniasis and effectively protected against vector-transmitted disease in endemic areas. However, the practice was discontinued due to major complications including non-healing skin lesions, exacerbation of skin diseases, and the potential impact of immunosuppression. Instead, tremendous effort has been made to develop killed, live attenuated, and non-living subunit formulations. Many of these formulations produce promising experimental results but have failed in field trials or against experimental challenge with infected sand flies. Recently, experimental models of leishmanization have unraveled the critical role of parasite persistence in maintaining the circulating CD4^+^ effector T cells responsible for mitigating the inflammatory response early after sand fly challenge and mediating protective immunity. Here, we put forward the notion that for effective vaccine design (especially non-living vaccines), the role of antigen persistence and pre-existing effector CD4^+^ T cells should be taken into consideration. We propose that dendritic cell-based vaccination strategies warrant greater attention because of their potential to act as long-term antigen depots, thereby emulating this critical requirement of naturally acquired protective immunity against infected sand fly challenge.

## Introduction to Leishmaniasis and *Leishmania* Vaccine Efforts

Leishmaniasis is a parasitic vector-borne disease caused by flagellated protozoans from the *Leishmania* genus. Disease occurs in multiple forms, including visceral disease that is fatal if left untreated and mucocutaneous and cutaneous forms that are associated with significant morbidity, including severe scarring even after clinical recovery. The *Leishmania* life cycle alternates between two developmental stages. The motile promastigote stage exists in the vector sandfly. Infected sandflies then deposit promastigotes into the mammalian skin together with infected sand fly-associated molecules while taking a blood meal. After transmission, promastigotes transform into non-motile amastigotes inside phagocytes of the mammalian host where they establish long-term chronic infection (1). Protection against infection is mainly associated with induction of parasite-specific T helper 1 (Th1) CD4^+^ T cells, and individuals with a healed but persistent primary cutaneous infection are protected against reinfection.

Prophylactic vaccination against leishmaniasis was initially performed by direct inoculation of live infectious parasites into naive individuals, a process referred to as leishmanization (2). Although very effective at inducing protective Th1 immunity, leishmanization has largely been discontinued due to safety concerns associated with administration of a live virulent organism, concerns which remain unresolved (3). To improve the safety prolife of a *Leishmania* vaccine, numerous killed, live attenuated, and non-living subunit formulations combined with Th1 adjuvants and delivery systems have been employed in an attempt to mimic the protective immunity generated by natural primary infection or leishmanization. These efforts have been extensively reviewed elsewhere (4–6). Briefly, starting in the 1940s, inoculation of killed *Leishmania* promastigotes was employed as a vaccine; however, low immunogenicity and poor protection hampered further use (7). Subsequently, genetic manipulation was employed to generate live but pathogenically attenuated parasites (8) and targeted gene manipulation of specific virulence-related genes resulted in numerous vaccine candidates (9, 10). Live attenuated parasites, although promising, are still viewed by some to harbor significant risk due to the potential of reversion back to the wild-type strain (11). In this regard, non-pathogenic *Leishmania* strains (such as *Leishmania tarentolae*), which are highly similar to pathogenic strains but lack virulence genes (12), have also been proposed as potential vaccine candidates (13).

In addition to whole pathogen approaches, subunit vaccines composed of subcellular components have drawn significant attention due to lower safety risks and a feasible production pipeline. Various formulations have been under intensive investigation to potentiate protein subunit vaccines including innovative adjuvants such as monophosphoryl lipid A (MPL) or glucopyranosyl lipid A (GLA), TLR4 agonists suitable for use in people (14); delivery systems including water-in-oil stable emulsions (SE) (15) or liposomes (16), DNA constructs delivered alone (17) or with a delivery system (18); dendritic cell (DC)-based vaccines (19); and even vectored vaccines (20, 21). Nonetheless, no effective subunit human vaccine is marketed despite promising experimental outcomes. The only vaccine formulation that has entered human clinical trials is Leish-F, a tri-fusion protein composed of TSA, *Lm*STI1, and LeIF. In MPL-SE formulation, the vaccine is safe and immunogenic in patients with cutaneous and mucocutaneous leishmaniasis as a therapeutic vaccine (22, 23). In GLA-SE formulation, it appears even more effective than MPL-SE (24). Other polyprotein vaccine formulations, including CPA-CPB-A2 (25, 26) and A2-Kmp11-CPB-SMT (KSAC) (27), have also shown promising results in experimental models and dogs.

## Leishmanization and the Role of Parasite Persistence in Naturally Acquired Immunity

A literature review on *Leishmania* vaccine history reveals that leishmanization (deliberate inoculation of live wild-type parasites without the disease exacerbating factors associated with sandfly bites) remains the most efficacious strategy to generate protective immunity against subsequent infected sandfly challenge both in field trials (28) and experimental models (29). Significant evidence suggests that this protection is lifelong (30). Traditionally, Leishmanization employed exudates from active lesions that were inoculated into a covered part of the body. Later on, live virulent promastigotes harvested from cell free cultures were used (31). Leishmanization with *Leishmania major* was practiced in the former USSR, Israel, and Iran (2, 32) but was discontinued due to loss of infectivity during continuous subculturing or freezing, rare complications at the inoculation site, and/or potential complications due to immunosuppression (i.e., a reduced response to diphtheria/pertussis/tetanus vaccination) (31). In Iran, non-healing cases further complicated the feasibility of the widespread use of Leishmanization as it was practiced in the 1980s (28, 33–35). While leishmanization is no longer widely practiced, the threat of leishmaniasis remains, and intensive investigation is under way to develop killed, naturally attenuated or genetically modified live parasites, or subunit vaccines that replicate the protection mediated by Leishmanization (36). However, evidence from both C57BL/6 and BALB/c experimental mouse models has shown that a key factor in the efficacy of leishmanization is the persistence of the parasite following inoculation (37–39). Persistent antigen presentation then drives concomitant T-cell immunity meaning that the protection against reinfection coincides with the persistence of the primary infection (40–42). Treatment of persistently infected mice to achieve sterile cure renders those mice susceptible to new infections (37–39, 43). In this review, we have tried to discuss the correlates of this concomitant immunity and their relevance to effective prophylactic vaccine design, with specific reference to DC-based vaccination strategies.

## Non-Living Vaccines Fail to Protect Against *L. major* Sandfly Challenge Compared to Leishmanization

An unresolved concern about *Leishmania* vaccination in the past was that protection against needle challenge in experimental animal models did not translate to protection when similar “first-generation” vaccine formulations were tested in human field trials employing natural sandfly transmission (7). Based upon these observations, Peters et al. investigated the effect of vector transmission on the efficacy of an ALM/CpG vaccine (autoclaved *L. major* plus CpG oligonucleotide) following needle versus sandfly challenge of *L. major* in mice. The experiments were designed to determine if vector transmission was a barrier to vaccine efficacy. Challenge was performed 12 weeks after the last booster dose of the vaccine or 16–20 weeks following leishmanization. Both ALM/CpG-vaccinated and leishmanized mice were protected against needle challenge with 5 × 10^3^ sandfly-derived parasites, although leishmanized mice had significantly lower parasite loads than ALM/CpG vaccinated mice. By contrast, following sandfly transmission the parasite number per each bitten ear of ALM/CpG vaccinated mice was comparable to age-matched non-vaccinated mice while leishmanized mice provided robust protection at 4 weeks post-challenge. Flow cytometry data analysis clearly detected CD4^+^/IFN-γ^+^ and CD4^+^/TNF-α^+^ T cells as early as 3 days post sandfly challenge only in leishmanized mice while the response in the vaccinated group was delayed until at least day 7 post-challenge. Of note was the higher frequency of cytokine-positive cells in the ALM/CpG-vaccinated group in response to needle versus sandfly challenge. The authors observed massive neutrophil recruitment 1-day post sandfly challenge that was maintained for at least 28 days, whereas neutrophil numbers rapidly declined to baseline within 3 days post needle challenge. Neutrophil depletion following the establishment of infection remarkably enhanced the potency of the immune response in ALM/CpG-vaccinated mice, resulting in comparable control of parasites as compared to leishmanized mice. They concluded that inflammatory conditions at the bite site actively compromise the effector function of the memory response generated by the killed ALM/CpG vaccine (29), possibly through modulation of antigen-presenting phagocytic cells (44).

In a second study by Peters et al., two well-known vaccine candidates, KSAC/GLA-SE and L110F/GLA-SE, were compared to ALM/CpG and leishmanization following needle or sandfly challenge of *L. major*. Again only leishmanized mice mounted a robust early immune response at 2 weeks post-challenge and potently controlled parasite burden at 4 weeks post needle challenge. Following needle challenge, polyprotein + GLA-SE-vaccinated mice reduced parasite loads to the same degree as mice vaccinated with ALM/CpG, but not comparable to the reduction observed in leishmanized mice. Following sandfly challenge, vaccinated groups once again failed to mount a comparable response observed in leishmanized mice and failed to provide protection, this time examined 6 weeks post sandfly challenge. Comparison of healed and vaccinated mice once again revealed negligible amounts of neutrophils at the dermal bite site in leishmanized, but not vaccinated, mice, corresponding to low parasite loads and a very high IFN-γ/IL-17 ratio following antigen-specific stimulation of dermal derived CD4^+^ T cells. They argued that a rapid and robust (high IFN-γ) immune response is the clearest correlate of effective immunity and is required to counteract the immune modulatory conditions at a sandfly bite site (45). Together these studies have turned attention to the difference between needle and sandfly challenge at early time points post infection. The early, bite-mediated, inflammatory response appears to influence infection outcome and must be considered when testing vaccines.

## Neutrophils Modulate Sites of Infection Early after Challenge

While phagocyte recruitment at sites of inflammation is one of the founding observations in immunology, Peters and Egen et al. employed the advantages of 2-Photon Intra Vital Microscopy to image the massive recruitment of neutrophils to a site of infection in the mouse ear epidermis. In this way they were able to record early *in vivo* events, within a few minutes after exposure of the dermis to *L. major*-infected sandfly bites (29, 46). These studies demonstrated that sandfly challenge quantitatively recruits more neutrophils than needle challenge and in a more sustained manner and that the majority of inoculated parasites infect and survive within neutrophils. Neutrophil depletion prior to challenge dramatically reduced both the parasite number per ear and the incidence of ears with detectable parasites, an observation that was also made early after needle challenge (47) or employing neutrophil deficient mice and *Leishmania mexicana* infection (48). They concluded that *L. major* exploits the innate host response to sandfly bite to establish disease, a concept originally proposed employing *in vitro* infection of neutrophils (49).

**Figure 1:**
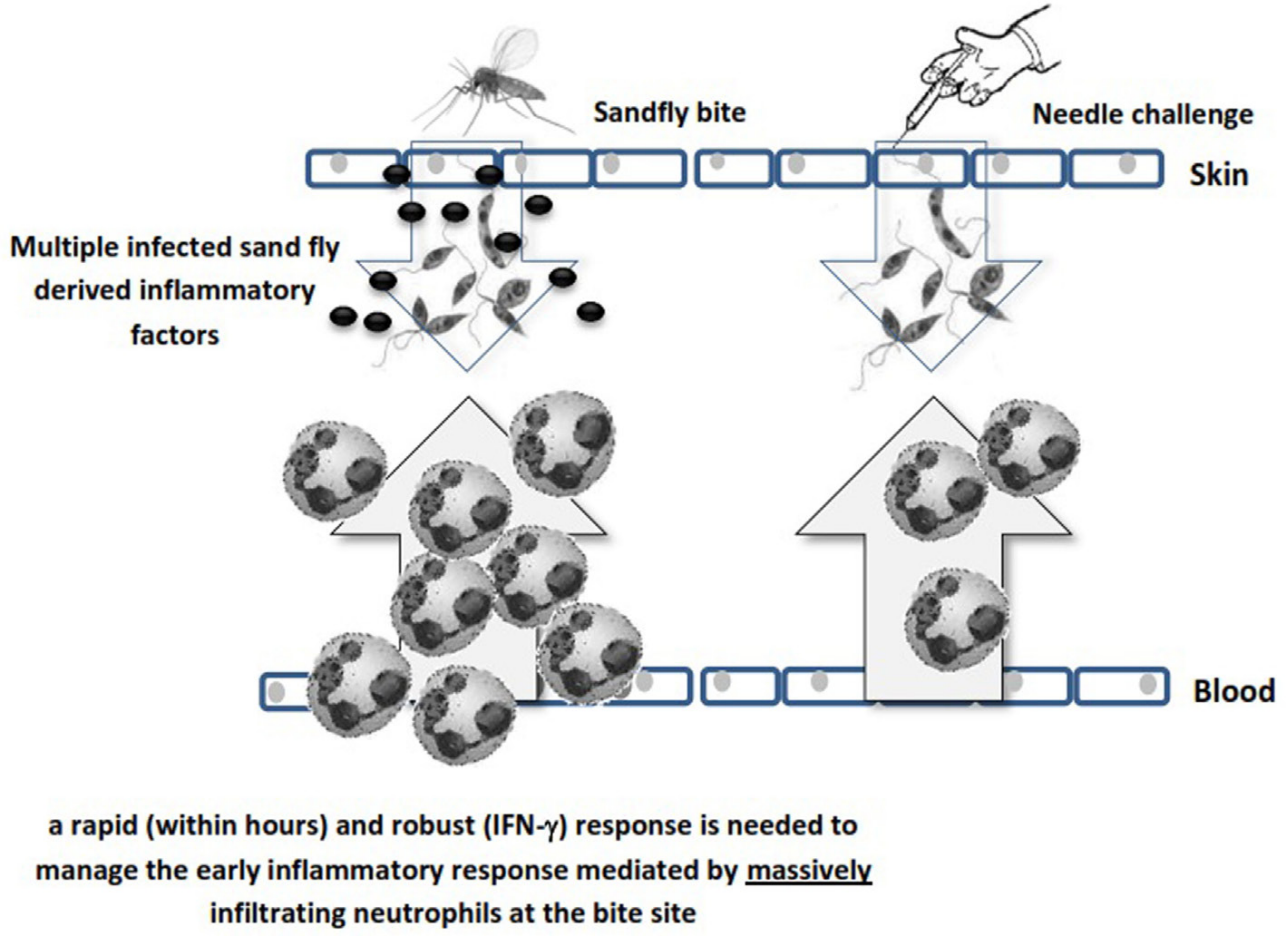
Schematic illustration of the difference between sandfly’s bite and needle challenge in neutrophil recruitment. Different sandfly and parasite mediators massively recruit neutrophils to the bite site while needle challenge is less reactive in this respect due to lack of saliva factors.

The enhanced neutrophil recruitment observed after sandfly bite is likely due to multiple inflammatory signals, some of which include trauma in the skin and blood vessels (either by needle or sandfly), salivary protein components (50), parasite secretory gel (51), *Leishmania*-derived exosomes (52), coinoculation of virus (53, 54), and more recently coinoculated bacteria (55). These factors can influence neutrophil recruitment to the bite site irrespective of the *Leishmania* parasite. Neutrophils populate the infection site before any other inflammatory cells and engulf parasites. Since *Leishmania* parasites are obligatory intracellular microorganisms, they benefit from the early neutrophil infiltration. However, neutrophils are equipped with different granules containing microbicidal compounds for killing invaders and can generate neutrophil extracellular traps (56). Therefore, some parasite species have evolved to modulate these defense mechanisms and to survive within neutrophils (46, 48, 49, 57). Neutrophil function is tightly regulated due to the hazardous materials they contain and within a few hours to days they undergo apoptosis. Phagocytic cells remove the remnants of apoptotic bodies (efferocytosis) and live parasites, a process associated with significant modulation of professional antigen-presenting cells (APCs) (44, 58). This early influence of neutrophils on sites of infected sandfly challenge implies that a rapid (within hours) and robust IFN-γ-mediated immune response is required to manage the innate immune response early after sandfly deposition of the parasite (Figure 1).

## Pre-Existing Effector T Cells Mediate Concomitant Immunity

**Figure 2:**
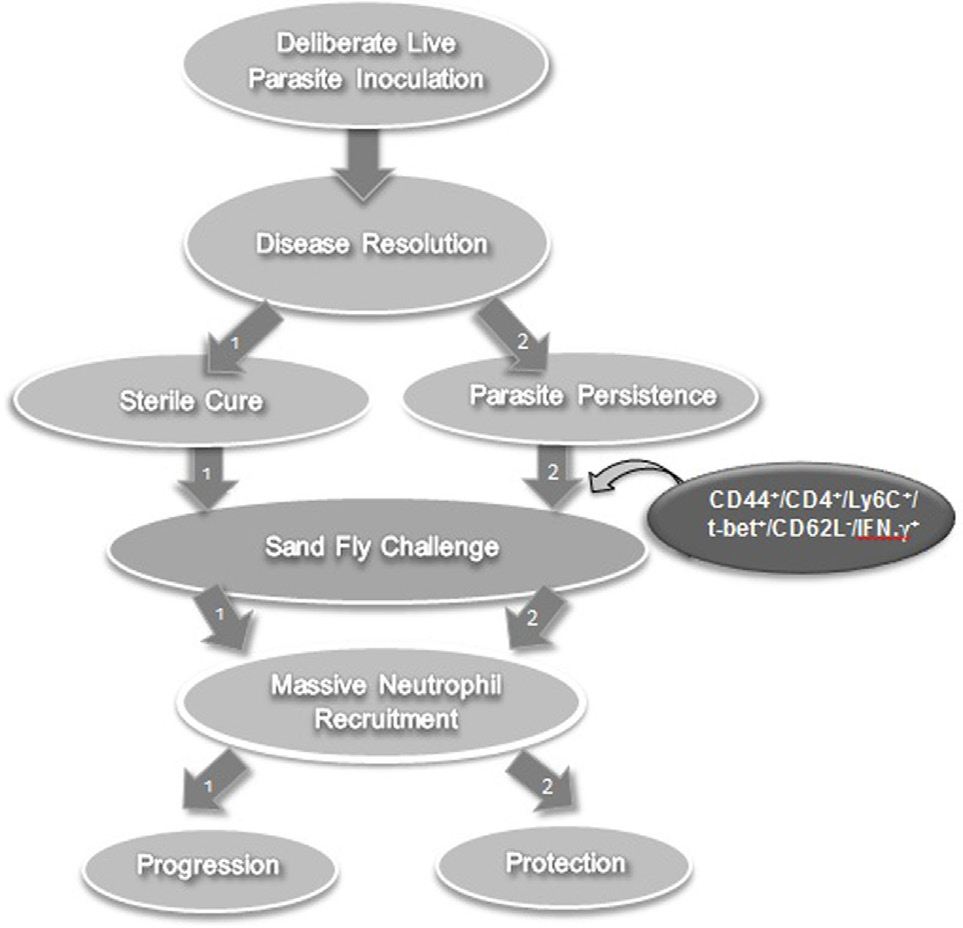
Lessons learned from leishmanization in experimental models. Leishmanization (or deliberate parasite inoculation) fails to protect against sandfly challenge in the absence of persistent parasite (path number 1). However, if the parasite persists after healing, it gives rise to a T effector cell population that mediates effective protection against sandfly challenge (path number 2).

The requirement for persisting infection to maintain concomitant immunity suggests that the phenotype of responding T cells may be highly diverse following leishmanization, consisting of both memory and effector subsets (39, 59). Peters et al. further characterized the nature of the rapidly recruited cells that correlated with concomitant immunity in resistant mice (59). To this end, they leishmanized C57BL/6 mice with 10^4^ *L. major* metacyclic promastigotes subcutaneously in the footpad and used these after complete healing of the lesions (leishmanized mice). They initially determined that CD4^+^ but not CD8^+^ T cells-mediated protective immunity upon adoptive transfer (from leishmanized mice to naïve mice) and challenge. Three days following exposure of the ear dermis of leishmanized mice to the bites of *L. major*-infected flies, dermal-derived CD3^+^CD4^+^ T cells were analyzed for cytokine production. These CD4^+^ T cells produced high levels of IFN-γ as examined by direct intracellular staining without antigen or pharmacological stimulation. CD4^+^IFN-γ single producers that rapidly populated the bite site as early as 20 h after *L. major* challenge in leishmanized mice (the earliest time point examined) lacked the proliferation marker Ki-67. This indicated that they are not derived from CD62L^+^ memory cells that have undergone proliferation after secondary challenge. The rapidly recruited IFN-γ-producing population could also be efficiently recruited to the site in an antigen non-specific manner by needle inoculation of PBS. To further confirm the effector nature of these cells, adoptive transfer system was used to enable tracking of CD4 T cells obtained from chronically infected mice to the dermal site of challenge in naïve recipients. Violet proliferation dye-labeled, Th1 marker (T-bet) enriched CD44^+^CD4^+^CD62L^+^ central memory T cells (T_CM_) and CD44^+^CD4^+^CD62L^−^ effector memory (T_EM_)/effector T (T_EFF_) populations isolated from healed mice were cotransferred into naïve mice and challenged 1 day after. On day 3 post-infection, adoptively transferred CD44^+^CD4^+^T-bet^+^CD62L^−^ cells were detected in the ear while CD44^+^CD4^+^T-bet^+^CD62L^+^ cells populated draining lymph nodes (dLNs) but not skin. On day 5 post-infection, the vast majority of antigen-specific IFN-γ^+^ T cells in the ear were derived from the CD44^+^CD4^+^T-bet^+^CD62L^−^ effector population that had not divided, while very few IFN-γ^+^CD44^+^CD4^+^T-bet^+^CD62L^+^ T cells were found, and those that were found had undergone proliferation. In contrast, on day 12, proliferated IFN-γ^+^ cells derived from the transferred CD44^+^CD4^+^T-bet^+^CD62L^+^ T_CM_ population dominated the dermal infiltrate. Remarkably, and in agreement with observations by Zaph et al. (39), only the CD44^+^CD4^+^T-bet^+^CD62L^−^ effector population mediated protective immunity at 3 weeks post-challenge. Therefore, the early recruitment of CD44^+^CD4^+^ IFN-γ^+^ non-dividing (Ki-67^−^) effector cells (CD62L^−^) correlated with protective immunity. Another marker, Ly6C, a marker of Th1 effector cells (60), further differentiated the effector cells (T_EFF_) from effector memory cells (T_EM_). At the site of challenge, the vast majority (≥70%) of IFN-γ making T cells in response to inoculated parasites highly express Ly6C. Of particular interest, employing a dermal challenge model of visceral infection with *Leishmania infantum* and i.v. labeling of circulating cells, Romano et al. were able to show that tissue infiltrating CD4^+^ T cells in the spleen and liver that are making IFN-γ *in situ* on day 3 post-challenge are also predominantly Ly6C^+^ cells and this correlated with protective immunity in the viscera (61). In contrast to effector memory T cells and T_CM_, antigen-specific Ly6C^+^CD44^+^CD4^+^T-bet^+^CD62L^−^ cells (T_EFF_) were remarkably prevalent in mice with a healed *L. major* infection but disappeared within 2 weeks of adoptive transfer from healed mice into naïve mice (devoid of parasite antigen). In contrast, adoptively transferred T_CM_ cells maintained their cell number over the same period, suggesting that Ly6C^+^ T_EFF_ cells are short-lived in the absence of antigen (59). Importantly, following adoptive transfer and challenge, Ly6C^+^ T_EFF_ cells emulated the protective response observed in intact leishmanized mice and mediated robust protection versus all other antigen-experienced Ly6C^+^CD44^+^CD4^+^ T cell subsets combined (59). More recently, Glennie et al. also demonstrated that circulating CD4^+^ T cells are required for optimal protection against infectious challenge, although the authors did not define which CD4^+^ subset mediated this protection (62). Therefore, it was concluded that conventional vaccines that generate long-lived memory T cells in the absence of persistent antigen are unlikely to protect against subsequent sandfly challenge because they do not maintain the Ly6C^+^ T_EFF_ population required to mediate the optimal response required to prevent disease infected sand fly challenge, despite mediating promising protection against experimental needle challenge. In contrast, the persistence of parasites following leishmanization maintains a population of T_EFF_ cells that mediate rapid and optimal immunity at the site of challenge (Figure 2). It should be noted that Ly6C is mouse specific and unlikely to be a useful marker in humans. It does, however, provide a useful phenotypic marker to track T_EFF_ cells in the mouse model to study the role of these cells in protective immunity.

## The Potential of DC-Based Vaccination Strategies to Address the Role of Antigen Persistence in Maintaining Protective Immunity

Dendritic cells are highly efficient APCs. They sense a large variety of signals in their environment, transport antigen from infected tissue to dLNs, express costimulatory molecules involved in naïve T cell activation and play a central role in the initiation of adaptive immunity (63). Moreover, these cells express various pathogen-associated pattern recognition receptors to drive Th1 or Th2 polarization (64). Moll et al. first reported that DCs at dLNs of healed C57BL/6 mice harbor persistent parasites (65, 66). Since then, DC-based vaccination has been under intensive investigation to induce protection against *Leishmania* challenge (67). Several pieces of evidence demonstrate that different DC types, once properly conditioned *ex vivo* with *Leishmania* antigens [either whole antigen (68–74), protein (75–79), peptides (19), or even plasmid DNA (80)] from dermotropic and viscerotropic parasites, mediate partial or complete protection against experimental needle challenge in animal models. Increased levels of IL-12 cytokine (77, 78), reduced T-cell derived IL-10 production (74), downmodulation of regulatory T cells, and TGF-β production (69) have all been associated with DC-induced, Th1-mediated, protection. In addition, vaccination with *Leishmania* antigen plus CpG-ODN pulsed DCs generates parasite-specific cytotoxic T lymphocytes that protected against visceral infection employing an intravenous needle challenge model (68).

While the use of *ex vivo* conditioned DC-based vaccines has significant practical limitations in the field (81), some have postulated that the protection mediated by *ex vivo* pulsed DC vaccination is actually mediated by recipient, not donor DCs. Schnitzer et al. demonstrated that fragments of antigen-loaded DCs and even DC-derived exosomes induce protection against *L. major* needle challenge 1 week following vaccination of BALB/c mice. The authors argued that non-living DC particles or cell-free exosomes are sufficient to confer protection in mice treated with antigen-loaded bone marrow-derived dendritic cells (BMDCs) *via* a Th1-polarized response. Although the underlying mechanism is not yet fully understood, DCs from recipient mice have been shown to actively take up the inoculated non-viable or cell-free particles. They postulated that a DC-targeted rather than a DC-based formulation could result in better outcomes (72).

Unlike other non-living vaccines, DC-based vaccine formulations have also shown promising results in maintaining protection for longer periods against needle challenge with *Leishmania*. Ramirez-Pineda et al. evaluated the potency and durability of the immune response after treating BALB/c mice with a single dose of *L. major* antigen-pulsed BMDC stimulated with CpG ODN (73). BALB/c mice were protected against needle challenge by a Th1-polarized immune response when challenged 1 or 16 weeks post-vaccination. The number of parasites was reduced ~10^5^-fold after short-term (1 week post-vaccination) or ~10^3^–10^4^ fold after long-term (16 weeks post-vaccination) challenge in mice pre-treated with CpG/Ag-loaded BMDC (compared to unprotected control mice at 5 weeks post-infection). Consistent with previous observations, IL-12 produced by LmAg-CpG-pulsed BMDCs is not a determining factor for Th1 deviation, instead recipient-derived IL-12 was required. Footpad swelling was also markedly reduced. Interestingly, when vaccinated mice that had recovered from the first challenge were rechallenged at week 10 post-primary infection (i.e., a second infectious challenge) and monitored for 10 more weeks, the parasite burden at the rechallenge site was again ~10^5^-fold lower than those of naive mice with negligible footpad swelling. Of note was the synergistic and long-lasting effect of one dose of LmAg-CpG-pulsed BMDC on the control of parasites at the site of challenge, 16 weeks after the last booster. Although not discussed in the paper, these results suggest that LmAg-CpG-pulsed BMDC vaccination combined with acute exposure to infection could provide long-term protective immunity to subsequent exposures without the confounding factors associated with leishmanization alone. In this case, parasite persistence at the site of the first challenge likely provides a source of antigen but without pathology and maintains T_EFF_ cells. Furthermore, DC vaccination has also protected against a challenge almost 16 weeks post-vaccination, an implication of long-lasting antigen presentation. The maintenance of T_EFF_ cell in this case still needs to be addressed.

The *ex vivo* loading approach is quite laborious and is not feasible for large-scale vaccination efforts. However, *in vivo* targeting approaches already practiced in viral and bacterial infections and cancers have generated new hope to reconsider DCs as potential vaccine targets against *Leishmania* infection, particularly with regard to antigen persistence and the maintenance of an effector T cell population. Matos et al. were the first to demonstrate the potency of a DC-targeted vaccine with *Lm*STI-1 as an antigen candidate and poly-ICLC as a DC stimulator. *Lm*STI-1 was directly targeted to DCs by fusion to a specific DC marker (DEC205). The results were promising since two doses of vaccine reduced parasite loads by several logs in BALB/c after both low-dose intradermal or high-dose subcutaneous *L. major* challenge and this coincided with markedly reduced pathology. Higher IFN-γ/IL-4 ratios and lower levels of IL-10 correlated well with the markedly reduced parasite burden both in dLNs and footpads at 12 weeks post-challenge. Of particular note was that DC-targeted vaccine maintained protective effect when low-dose intradermal challenge was delayed to 12 weeks after the last booster, demonstrating significant vaccine durability (79). While these studies did not assess the critical cells responsible for protection, these data raise the potential that DCs in this setting are acting as long-term antigen depots and maintaining a population and highly protective T_EFF_ cells prior to challenge.

While protein antigens are thought to be relatively short-lived *in vivo*, recent observations have suggested that follicular dendritic cells (FDCs) can act as long-term APCs, suggesting they can act as antigen depots. Recently, Heesters et al. have postulated that FDCs are able to internalize foreign antigens within non-degradative endosomes and keep them intact for further presentation to B cells (82). This provides a clue as to why DCs are able to preserve antigen for long periods. Although experimental evidence is lacking, FDCs are estimated to recycle antigen for 1 year or even more (83). Heesters’s group has confirmed antigen persistence up to 3 months following antigen injection (82). Based upon the observations obtained following delayed challenge of the DEC205 DC-targeted vaccine, DCs may contribute to concomitant immunity by maintaining depots of protein antigen. However, further evidence needs to be generated regarding the capacity of DCs to act as long-term antigen depots and the subsets of antigen-specific T cells maintained under these conditions. Moreover, a more precise definition of which DC subsets are involved, including a better definition of the relative contributions of cDC1, cDC2, and FDCs (84), and which markers, in addition to DEC205, are ideal to target with antigen delivery systems is required to develop an effective vaccine formulation.

The dual role played by DCs as immunomodulators of adaptive immunity and their potential as antigen depots are also relevant for live attenuated vaccine. Genetically modified non-virulent parasites and naturally attenuated parasites are thought to undergo the same infectious process as their wild-type counterparts (8). Therefore, persistent non-virulent parasites, including those subjected to genetic modifications, should be ideal surrogates of live virulent parasites (85). If attenuated parasites survive after vaccination, they may provide a sufficient amount of parasite antigens to maintain T_EFF_-dependent long-term protective immunity. Remarkably, reduced persistence of attenuated parasites, long considered a substantial pre-requisite to address safety concerns (86), may in fact lower the long-term protective efficacy of live attenuated parasite against naturally infected sandfly challenge.

## Canine Vaccines and Their Implications for Human Vaccination

In contrast to observation in people, subunit vaccines have been met with some success in canines. Leishmune^®^ is a vaccine that includes the *Leishmania donovani* glycoprotein fraction known as fucose–mannose ligand with QS21 as adjuvant providing 92–95% protection and 76–80% of efficacy in endemic areas of Brazil (87–89). The immune response raised by the vaccine is long-lasting (about 3.5 years) (90) and blocks the transmission of the parasite back to sand flies. While the effector or memory phenotype of responding T cells or the persistence of parasites or parasite antigen has not been analyzed in the context of canine infection, both Ab- and cell-mediated immune responses appeared to benefit from natural boosting in endemic areas. Therefore, early and continued exposure of Leishmune^®^-vaccinated dogs in endemic areas to infected sand fly bites may well explain the efficacy of the vaccines, as natural boosting would provide the parasite antigen required to maintain the protective T_EFF_ immune response. Whether a similar phenomenon could be expected to occur in people, who are likely less exposed to sand fly bites, is unknown. Of interest, it is speculated that the glycoprotein antigen included in this vaccine, with about 52.3% mannose content (91), is effectively taken up by mannose receptors on DCs (92). Therefore, effective delivery of Leishmune^®^ to DCs combined with early and continued natural boosting may generate and maintain effector T cells (T_EFF_ cells), resulting in protection against sand fly transmitted disease which needs to further addressed.

Another registered formulation such as Leish-Tec^®^ in Brazil is composed of recombinant A2 antigen plus saponin (93). This formulation conferred about 40% protection against infection by artificial intravenous injection of high-load *L. infantum* promastigotes (94). A double-blinded, randomized phase III trial was conducted to test the efficacy of Leish-Tec^®^ in an endemic area in Brazil. A large number of outbred healthy dogs were followed up to 1 year, 96% of the vaccinated dogs remained uninfected. Parasitological diagnostic results, confirmed 71% protective effect of the vaccine in this population (95). A comparative trial in dogs vaccinated with Leishmune^®^ or Leish-Tec^®^ showed no significant differences with regards to clinical and parasitological aspects, IgG seropositivity, or dog infectiousness (96). These results are relatively in compliance with our concept but there is no evidence yet to prove this, and further investigation is necessary and quite worthwhile due to the long-term effect of these vaccines.

## Conclusion

This review presents evidence that suggests non-living vaccines are at risk of failure to protect against sandfly transmission of *Leishmania* parasite if they do not provide persistent antigen. The requirement for prolonged antigen presentation appears to be due to the fact that the CD4^+^ T cells mediating the optimal protective immune response against natural sand fly challenge are effector T cells that pre-exist challenge, are short-lived in the absence of antigen, and which mitigate the early innate response after *L. major* sandfly challenge in leishmanized C57BL/6 mice. If specific subsets of DCs can act as long-term natural depots of antigen, then DC-targeted vaccine formulations could be more promising than other types of non-living formulations that do not target these DC populations. It is tempting to speculate the role of T_EFF_ cells in lifelong protection against sand fly challenge for *Leishmania* species other than *L. major*, as suggested by recent observations employing visceral infection model with *L. infantum* (61). Furthermore, to properly evaluate experimental vaccine efficacy, needle challenges should be replaced with models that either employ natural sand fly challenge or sufficiently incorporate findings from natural transmission in the interpretation of the results.

## Author Contributions

NS: substantial contributions to the conception or design of the work. SR and NP: revising the manuscript critically for important intellectual content and agreement to be accountable for all aspects. NS, SR, and NP: final approval of the version to be published.

## Conflict of Interest Statement

The authors declare that the research was conducted in the absence of any commercial or financial relationships that could be construed as a potential conflict of interest.
